# Glutathione Peroxidase 4 is associated with Neuromelanin in Substantia Nigra and Dystrophic Axons in Putamen of Parkinson's brain

**DOI:** 10.1186/1750-1326-6-8

**Published:** 2011-01-21

**Authors:** Frederick P Bellinger, Miyoko T Bellinger, Lucia A Seale, Andrea S Takemoto, Arjun V Raman, Takanori Miki, Amy B Manning-Boğ, Marla J Berry, Lon R White, G Webster Ross

**Affiliations:** 1 Cell and Molecular Biology Department, John A. Burns School of Medicine, University of Hawaii, Honolulu, HI 96813 USA; 2 Kuakini Medical Center, Honolulu, HI 96817 USA; 3 Veterans Affairs Pacific Islands Health Care System in Honolulu, Honolulu, HI; 4 Department of Anatomy, University of Kagawa, Saiwai, Takamatsu, 760-8521 Japan; 5 Center for Health Sciences, SRI International, Menlo Park, CA 94025 USA

## Abstract

**Background:**

Parkinson's disease is a neurodegenerative disorder characterized pathologically by the loss of nigrostriatal dopamine neurons that project from the substantia nigra in the midbrain to the putamen and caudate nuclei, leading to the clinical features of bradykinesia, rigidity, and rest tremor. Oxidative stress from oxidized dopamine and related compounds may contribute to the degeneration characteristic of this disease.

**Results:**

To investigate a possible role of the phospholipid hydroperoxidase glutathione peroxidase 4 (GPX4) in protection from oxidative stress, we investigated GPX4 expression in postmortem human brain tissue from individuals with and without Parkinson's disease. In both control and Parkinson's samples, GPX4 was found in dopaminergic nigral neurons colocalized with neuromelanin. Overall GPX4 was significantly reduced in substantia nigra in Parkinson's vs. control subjects, but was increased relative to the cell density of surviving nigral cells. In putamen, GPX4 was concentrated within dystrophic dopaminergic axons in Parkinson's subjects, although overall levels of GPX4 were not significantly different compared to control putamen.

**Conclusions:**

This study demonstrates an up-regulation of GPX4 in neurons of substantia nigra and association of this protein with dystrophic axons in striatum of Parkinson's brain, indicating a possible neuroprotective role. Additionally, our findings suggest this enzyme may contribute to the production of neuromelanin.

## Background

Parkinson's disease (PD) is characterized pathologically by the development of cellular inclusions called Lewy bodies composed of aggregated proteins, primarily α-synuclein (AS) as well as ubiquitin and other misfolded proteins. The presence of ubiquitin within these structures suggests a build-up of proteins that were directed towards proteolytic degradation. AS also accumulates in the dopaminergic projections from these neurons forming dystrophic axons. PD is associated with severe loss of dopamine (DA) neurons in the substantia nigra (SN) and their striatal terminals, leading to dopamine depletion and consequential motor impairments. Accumulation of oxidized biomolecules associated with degeneration of DA neurons indicates the involvement of oxidative stress mechanisms [1], and the highly oxidizable aromatic structure of DA and its metabolites are thought to contribute to these processes [2].

Neurons in SN pigmented with neuromelanin are particularly sensitive to neurodegeneration in PD [3]. With aging, SN neurons in primates gradually transition from dopaminergic neurons, identified by the presence of the DA-synthesizing enzyme tyrosine hydroxylase (TH), to dopaminergic neurons expressing neuromelanin and eventually to cells that do not synthesize dopamine (TH-negative) but have neuromelanin [4]. The formation is postulated to require hydroperoxidase activity. Peroxidase activity associated with neuromelanin has been demonstrated and is increased in postmortem PD brain [5]. However, a hydroperoxidase responsible for neuromelanin formation has not specifically been identified.

The glutathione peroxidases (GPX) are hydroperoxidases essential for maintaining redox balance in cells [6]. Most of the GPX enzymes are selenoproteins that contain the micronutrient selenium (Se) incorporated as the amino acid, selenocysteine [7]. Se deficiency results in impairments in neurological function [8], highlighting the importance in brain of proteins utilizing this essential trace element. GPXs have been implicated in PD as brain glutathione levels are decreased in early stages of PD [9]. As glutathione is a cofactor for the GPX enzymes, its loss results in decreased peroxidase activity [10]. Overexpression of GPX1 attenuates 6-OHDA induced toxicity to dopamine neurons in rodent models of PD [11,12]. A recent study showed that GPX1 is present in human microglia and may play a role in removing AS aggregates from neuron terminals [13].

Less is known about the possible role of GPX4 in PD. Whereas GPX1 reduces inorganic hydrogen peroxide, GPX4 is the major enzyme that reduces membrane lipid peroxides [7]. Recent studies indicate the importance of the phospholipid hydroperoxidase GPX4 in the brain. Genetic deletion of GPX4 is embryonic lethal [14,15], and conditional deletion of GPX4 in brain results in severe neurodegeneration [16], indicating that the role of GPX4 in lipid hydroperoxide removal is essential for cell viability. Translation of the GPX4 protein is regulated by DJ-1, a protein implicated in a recessive form of early-onset PD [17]. Oxidation of DJ-1 is associated with an increase in cerebral cortex of GPX4 in subjects with sporadic Parkinson's disease [18]. However, as expression of GPX4 has not been investigated in the brain regions most affected in PD, i.e. the substantia nigra (SN) and basal ganglia, it is unclear if this upregulation is related to the etiology of PD or secondary to PD pathology. Lastly, the phospholipid hydroperoxidase function of GPX4 makes it a candidate enzyme for neuromelanin formation.

Here we evaluate the pattern of expression of GPX4 in the nigrostriatal pathway and whether expression is altered in PD as a possible response to oxidative stress. We report that GPX4 is co-localized with neuromelanin in SN dopamine neurons, decreased in PD midbrain, and associated with dystrophic axons in PD putamen.

## Results

We examined GPX4 in postmortem brain of 12 subjects that had been clinically diagnosed with PD, as well as 11 subjects without clinical or postmortem pathological features of PD. Tissue was obtained from the Honolulu-Asia Aging Study (HAAS). Data for all subjects are summarized in Table 1. There were no significant differences in age or post-mortem interval (PMI) between control and PD subjects. However, data obtained at autopsy demonstrated that the density of cells in SN was reduced in PD subjects to about a third of controls. The degeneration was most severe in lateral ventral SN, where the cell density in PD subjects was approximately a fifth of control subjects [19].

**Table 1:** Study Subjects.

	Control (*n = 11)*	PD (*n = 12*)
Age Range (YR):	76.6-91.7	79.6-92.9
Age at Death (YR):	84.0 ± 1.3	84.2 ± 1.0
Post-Mortem Interval (HR):	17.01 ± 2.7	9.57 ± 4.1
Median (and Range) Braak stage:	0 (0)	5.5 (5-6)
Total SN Cell Density:	21.7 ± 1.9	8.2 ± 1.4^†^
Lateral-Ventral SN Cell Density:	27.6 ± 3.3	5.2 ± 1.6^†^

† *indicates significantly different from controls (P < 0.0001).*

HAAS subject information obtained at autopsy. Methods published previously [19].

All decedents were American-born males of Japanese ancestry living in Hawaii. Data are mean ± SEM unless indicated otherwise.

### GPX4 in SN

**Figure 1:**
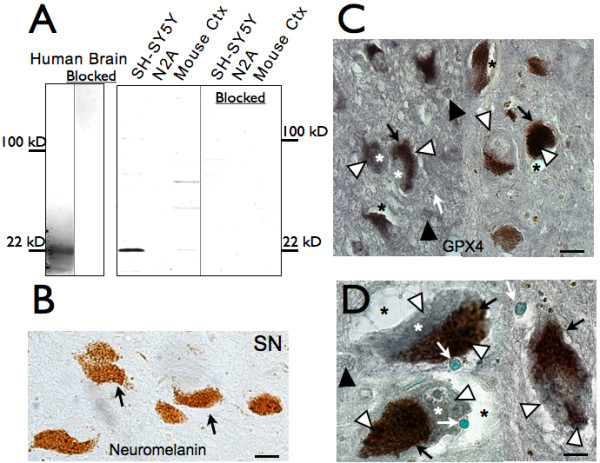
**GPX4 is associated with neuromelanin in SN neurons**. GPX4 colocalizes with neuromelanin in SN of control brain. A. Western blot showing antibody specificity in human brain (left) and human and mouse neuroblastoma cells and mouse cortex (right). For "blocked" controls, antibody was preabsorbed with original recombinant antigen. B. Immunohistochemistry negative control with primary antibody omitted. Brown pigment is endogenous neuromelanin (black arrows). C. Robust GPX4 expression (dark grey Ni-DAB, white arrowheads) is associated with neuromelanin (brown, black arrows) and in the cytoplasm (*). D. Enlarged cell images, counterstained with methyl green to show nuclei of these neurons (white arrows). Scale bars: B, C, 20 μm, D, 10 μm.

We tested GPX4 antibody specificity by western blot. As shown in Figure 1A, the antibody reacted with a 22 kD band in protein from human brain and from SH-SY5Y human neuroblastoma cells. This immunoreactivity was blocked by preabsorbing the antibody with the recombinant GPX4 antigen. However, the antibody did not recognize protein in extracts from the mouse N2A neuroblastoma cell line, and reacted with additional bands in mouse cortex protein. Thus the antibody appeared to be very specific for GPX4 in human brain but not in mouse cells and tissue.

We examined subcellular localization of GPX4 in SN in control brain using immunohistochemistry. GPX4 was mostly colocalized with neuromelanin in SN neurons (Figure 1C). SN neuromelanin can be clearly seen when primary antibody is omitted (Figure 1B). GPX4 immunolabeling with 3, 3-diaminobenzidine hydrochloride (DAB) containing NiCl_2_, in order to contrast with neuromelanin, coincided mostly within neuromelanin-positive neurons, and surprisingly was mostly associated with intracellular neuromelanin (Figure 1C, D). Weaker labeling of other cytoplasmic areas in these neurons is also present (shown by white asterisks), but some cytoplasmic regions were void of GPX4 immunoreactivity and appeared as empty space (black asterisks). Co-localization of GPX4 with neuromelanin was observed in midbrain sections from all 11 control and 12 PD subjects, although neuromelanin was notably reduced in SN of PD midbrain, presumably due to loss of neuromelanin-containing neurons. GPX4 labeling was also present at low levels in tissue surrounding neurons, in smaller glia cells (black arrowheads) and in midbrain white matter fibers (not shown).

**Figure 2:**
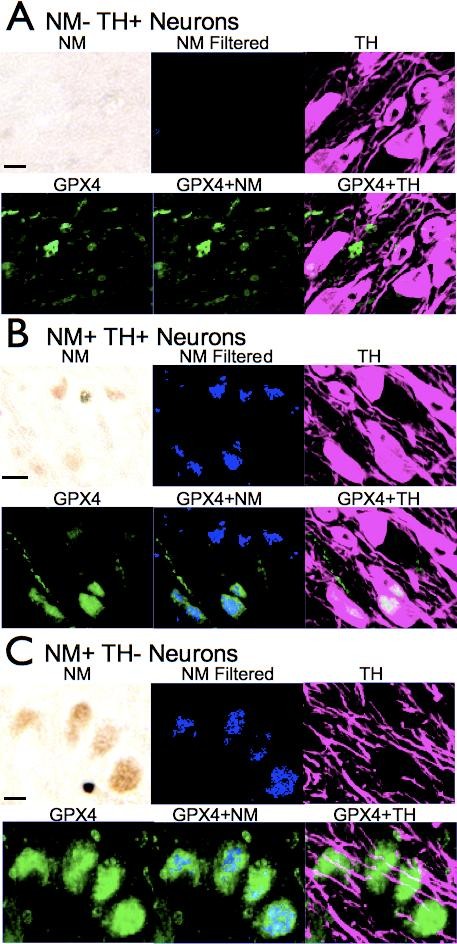
**GPX4 is found primarily in Neuromelanin-expressing neurons of SN**. GPX4 immunoreactivity is present in most cells expressing neuromelanin but is absent in many cells expressing TH but not neuromelanin. A. Example of cells expressing TH but not neuromelanin. Light microscope images (above left) were filtered for neuromelanin (blue, above center) to compare with TH immunoreactivity (magenta, above right) and GPX4 immunoreactivity (green, below left). GPX4 images combined with neuromelanin (below, middle) and TH immunoreactivity (below, right) are also shown for comparison. GPX4 is present in surrounding glia but absent in TH-expressing neurons. B. Examples of TH and neuromelanin expressing neurons. GPX4 is present in most cells with neuromelanin. C. GPX4 immunoreactivity is prominent in neurons containing neuromelanin but no longer expressing TH. (Note: as images were not taken with confocal microscopy, color changes only show only co-localization of area and not intracellular co-localization). Scale bars: 20 μm.

We used spectral imaging to compare GPX4 immunoreactivity in SN cells with presence or absence of neuromelanin. Figure 2A depicts a group of TH-positive cells that are negative for neuromelanin. GPX4 immunoreactivity is largely absent in these cells, although it can be seen in surrounding glia and white matter. Figure 2B shows a group of TH- and neuromelanin-positive neurons. GPX4 immunoreactivity was visible in most TH-positive cells expressing neuromelanin, although expression levels varied from cell to cell. Figure 2C shows a group of TH-negative, neuromelanin-positive cells. GPX4 labeling was strongest within these cells. These findings suggest GPX4 expression increases in SN neurons with the development of neuromelanin and continues to increase as expression of TH is lost.

### GPX4 in PD SN

**Figure 3:**
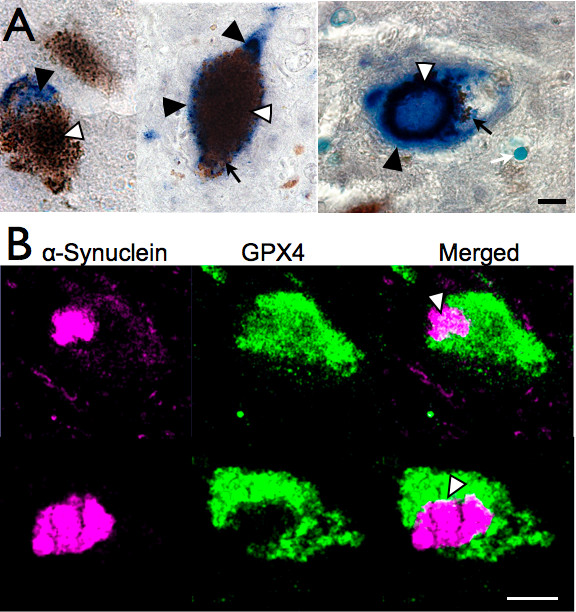
**GPX4 in nigral inclusions of PD brain**. A. GPX4 expression (dark grey Ni-DAB, white arrowheads) coincides with AS-positive Lewy bodies (blue BCIP, marked by black arrowheads) in SN. Black arrows indicate neuromelanin. B. Confocal microscope images of two cells showing relationship of GPX4 (green) to AS (magenta). The above example shows some co-localization of GPX4 with AS within a Lewy body, while the below example shows GPX4 only around the Lewy body perimeter. Scale bars: A, 20 μm, B, 10 μm.

We examined GPX4 immunoreactivity in SN of PD and control brain and its relation to Lewy bodies using an antibody to alpha synuclein. We found GPX4 associated with AS-positive Lewy bodies (Figure 3A), which were often found within or bordering cellular regions of GPX4/neuromelanin. This is in agreement with the association of GPX4 with neuromelanin, as Lewy bodies have some association with neurons that have high neuromelanin content [3]. Confocal sections though tissue with a thickness of 0.9 μm showed that GPX4 labeling present within Lewy bodies (3B, above), although generally less than in surrounding cytoplasm and typically confined to the perimeter of the inclusions (3B, below).

**Figure 4:**
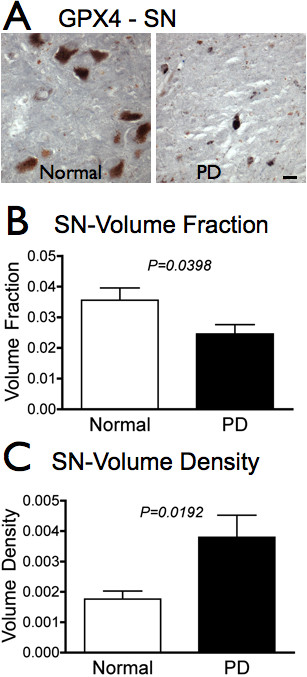
**GPX4 is reduced overall but increased relative to cell density in PD SN**. A. GPX4 (dark grey) is visibly reduced in SN of PD subjects (*n = 12*) compared to controls (*n = 11*). B. Total immunoreactivity of GPX4 is significantly reduced in SN of PD subjects compared to controls (P = 0.0398, Student's t-test). C. GPX4 immunoreactivity is increased relative to cell density (P = 0.0192, Student's t-test). Scale bars: 20 μm.

We investigated possible changes in GPX4 in PD brain by measuring area of immunoreactivity using unbiased stereology. Using a Cavalieri probe, we determined that GPX4 was significantly decreased in SN of PD brain compared with control SN (Figure 4A, B). The total volume fraction of GPX4 immunoreactivity in this region was reduced by nearly one third in PD brain, from 0.036 ± 0.004 in control SN (n = 11) to 0.024 ± 0.003 in PD SN (n = 12).

The reduction in total GPX4 immunoreactivity could be due to the severe cell loss in this region. In order to determine if loss of GPX4 could be explained by cell death, we investigated the volume of GPX4 as a function of cell density. We found that GPX4 immunoreactivity is actually *increased *relative to the cell density of surviving SN neurons (Figure 4C). GPX4 volume density is increased from 0.0018 ± 0.0002 in controls (n = 11) to 0.0038 ± 0.0007 in PD (n = 12) (P = 0.0192), indicating that GPX4 is either upregulated in surviving neurons or that a greater percentage of cells not expressing GPX4 are lost compared to cells with GPX4. This suggests a neuroprotective role for GPX4 within these neurons.

### GPX4 in Putamen

**Figure 5:**
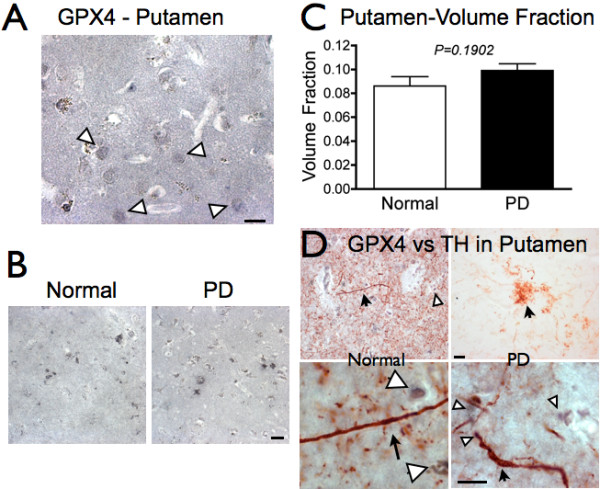
**GPX4 expression in putamen**. A. GPX4 immunoreactivity is detectable in cell bodies in the putamen of control brains (white arrowheads). B. Comparison of GPX4 labeling in normal and PD putamen. C. Mean GPX4 volume fraction is higher in PD (*n = 12*) putamen compared to control putamen (*n = 11*), although the difference is not significant (P > 0.05). D. Images of TH (Nova Red, red color, marked by black arrows) and GPX4 (DAB-Ni, blue/gray, white arrowheads) in putamen of non-PD (left) and PD (right) brain. Occasional swollen, dystrophic axons are present among the relatively few TH-positive fibers and terminals in PD putamen. Scale bars: 20 μm.

In putamen, GPX4 is associated with small cell bodies reminiscent of small stellate neurons or glia (Figure 5A). GPX4 immunoreactivity was not significantly different between normal and PD putamen (Figure 5B). The area of GPX4 immunoreactivity was slightly increased in PD putamen compared to controls, but the increase was not significant (Figure 5C). As PD-associated neurodegeneration is specific to DA axons from SN and not cell bodies in putamen, this finding supports a loss of GPX that is specific within DA neurons.

To determine if GPX4 was found in DA terminals, we used double-labeling immunohistochemistry with GPX4 and an antibody to tyrosine hydroxylase (TH). In non-PD putamen sections, GPX4 immunoreactivity (blue/gray) was generally not associated with TH-positive (red) fibers in control brain (Figure 5C, left), although weaker levels of GPX4 immunoreactivity are possible. Instead, GPX4 was mostly associated with smaller cells within putamen that are likely to be stellate neurons or glial cells. PD putamen contained relatively few TH-positive fibers and terminals, and some of the TH-positive axons were varicose and truncated (5D, right). Some GPX4 immunoreactivity was associated with the dystrophic axons in PD putamen (Figure 5D, lower right), in contrast with the lack of GPX4 found in TH-positive terminals in non-PD subjects.

**Figure 6:**
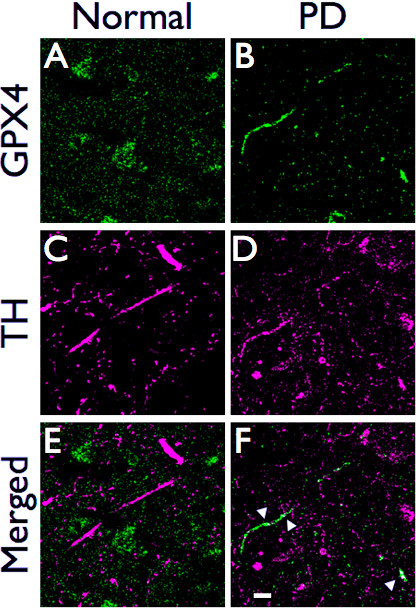
**GPX4 is associated with dystrophic axons in PD putamen**. Confocal images of GPX4 (blue) and TH (magenta) in non-PD (and PD brain (F, H, J). GPX4 has little association with TH in control brain sections, but colocalizes with TH in dystrophic axons in PD brain (shown by white color, white arrowheads). Scale bar: 10 μm.

We used confocal microscopy to further examine the relationship between GPX4 and TH in putamen axons (Figure 6). As described for light microscopy, we found little colocalization of GPX4 with TH in putamen samples from non-PD subjects. However, we found GPX4 concentrated in dystrophic axons and colocalization of GPX4 with TH in axonal varicosities (shown by white arrowheads). This suggests an upregulation of GPX4 in degenerating dopaminergic terminals.

Altogether, these findings indicate changes in GPX4 in SN and putamen that coincide with PD pathology, suggesting a role for GPX4 in the development of this disorder.

## Discussion

In this study, we have determined that altered GPX4 levels and distribution are associated with pathological PD changes. Specifically, overall GPX4 is greatly reduced in the SN of PD vs. control subjects, but when cell loss is taken into account, GPX4 is increased relative to cell density. Interestingly, GPX4 immunoreactivity is associated with neuromelanin in SN and colocalizes with AS-positive nigral Lewy bodies and dystrophic TH-positive fibers in putamen. These findings indicate either an upregulation of GPX4 in response to PD pathology, or an increase in survival of cells expressing GPX4, and further suggest a neuroprotective role of GPX4 in PD pathology.

GPX4 has recently been shown to prevent 12/15-lipoxygenase-dependent apoptotic cell death resulting from oxidative stress [16]. In the absence of GPX4, lipid peroxidation via 12/15-lipoxygenase leads to activation of the apoptosis-inducing factor (AIF) to induce cell death. Thus GPX4 is an important factor to prevent neuronal death from the increased lipid peroxidation found in PD neurons. However, GPX4 may be less effective in PD brain as glutathione levels are reduced early in the disorder [9]. This could lead to overproduction of GPX4 without the benefit of reduced oxidative stress and lead to its association with pathological structures.

Colocalization of GPX4 with neuromelanin is particularly suggestive of a role for GPX4 in aging of DA neurons. As GPX4 is a lipid hydroperoxidase [20], colocalization with neuromelanin suggests GPX4 could be a hydroperoxidase contributing to the synthesis of neuromelanin. The function of neuromelanin is currently unknown, but it is relevant that the substance accumulates in the SN of aging primates [4,21]. Neuromelanin is specific to catecholaminergic neurons of higher mammals, and SN neuromelanin is comprised of repeats of quinone structures which are likely formed from oxidized dopamine as well as dopamine synthesis or breakdown structures such as L-DOPA and DOPAC [21-23]. In a previous proteomics study, GPX4 was identified as a protein associated with neuromelanin in human brain [24]. Buildup of neuromelanin may be toxic to cells [25], although there is some evidence that it has a protective role in these neurons [26]. The colocalization of GPX4 with neuromelanin could indicate a response to oxidized lipids associated with neuromelanin. As neuromelanin is made up of lipids, protein and fatty acids, GPX4 may associate with neuromelanin in order to reduce the associated oxidized molecules. Conversely, an association of GPX4 with neuromelanin may result in a buildup of oxidized glutathione, further depleting the active pool of glutathione in PD.

The association of GPX4 with TH-positive degenerating axons in putamen suggests an up-regulation of GPX4 at the level of the putamen. A buildup of oxidized dopamine in axons could possibly induce expression of GPX4 to mitigate oxidative stress. An increase in GPX4, specific to dystrophic axons, could explain the apparent increase in GPX4 in putamen, which may be masked by a lack of change in cell bodies of neurons intrinsic to putamen. Furthermore, it seems possible that the changes in GPX4 in dystrophic neurons could be related to the colocalization of GPX4 with neuromelanin. Up-regulated GPX4 may accumulate in cell bodies, or may be transported from fibers as axons retract. The increase in GPX4 in soma may catalyze formation of neuromelanin from increased dopamine quinone in these neurons. Thus GPX4 may have an important role in the development of PD pathology.

## Conclusions

**Figure 7:**
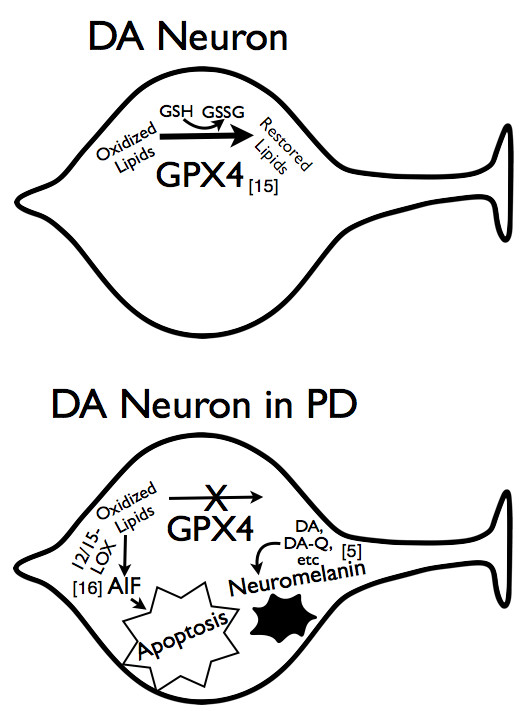
**Possible Role of GPX4 in Prevention of Apoptotic Cell Death and Production of Neuromelanin**. In healthy DA neurons, GPX4 functions to reduce oxidized lipids by oxidizing glutathione (GSH) to glutathione disulfide (GSSG) (above). However, a buildup of oxidized dopamine metabolites or a reduction of glutathione may lead to the synthesis of neuromelanin by GPX4, a process that could compete with reduction of oxidized lipids (below). The accumulated oxidized lipids will be metabolized by 12/15-lipoxygenase (12/15-lipoxy) and may eventually induce AIF and promote apoptotic cell death of DA neurons.

We have developed a model of the possible competing reactions of GPX4 in reduction of lipids and production of neuromelanin, depicted in Figure 7. GPX4 uses glutathione as a substrate to reduce oxidized lipids (above) [15]. However, a build-up of oxidized forms of DA-related compounds such as DA-quinones may drive the synthesis of neuromelanin from these compounds, a process that may compete with reduction of lipids (below) [21-23]. Depletion of glutathione and upregulation of GPX4 peroxidase activity in response to oxidative stress may further drive the production of neuromelanin and inhibit reduction of lipids [5]. Accumulated oxidized lipids are metabolized by 12/15-lipoxygenase, and metabolite products activate AIF to promote apoptotic cell death [16]. Thus under pathological conditions such as extreme levels of oxidation, the important protective properties of GPX4 may be redirected towards neuromelanin production, and this process may contribute to the loss of DA neurons in PD. Overall, our findings indicate an important neuroprotective role for GPX4 in neurons of the nigrostriatal pathway, and also suggest a role in neuromelanin synthesis associated with primate aging.

## Methods

### Subjects

Formalin-fixed human brain tissue was provided by the Honolulu-Asia Aging Study (HAAS), an ongoing longitudinal epidemiological study that has monitored the health and lifestyle of a cohort of Japanese-American men born between 1900 and 1919 and residing on Oahu, Hawaii [27]. Sections (10 μm) of SN and putamen from 12 subjects clinically diagnosed with PD and having marked pathological features including Lewy bodies and degeneration of dopaminergic terminals and cell bodies were examined. Sections from 11 age-matched subjects that had no symptoms of PD during life and no PD-associated neuropathology were used as controls in this study.

### Western blot

Post-mortem tissue from human occipital cortex was homogenized by sonication in 10 mM Tris/1 mM EDTA/protease inhibitor cocktail (1:1000; Sigma, St. Louis, MO), centrifuged at 1,000 × g for 10 min, and protein concentration in the supernatant was measured using Pierce BCA kit (Rockford, IL). Proteins were separated by SDS-PAGE and transferred to nitrocellulose. Protein was extracted from SH-SY5Y cells, N2A cells and mouse cortex using CellLytic buffer (Sigma) per manufacturer's instructions, separated by electrophoresis and blotted to PVDF membranes. Blots were blocked with Odyssey blocking buffer (LiCore Biosciences) for 1 hr and then incubated in GPX4 antibody (AbFrontier) diluted 1:2000. To confirm antibody specificity, antibody blocked with 50 μg/ml recombinant GPX4 antigen (AbFrontier) was also used. After washing with PBS containing 0.05% tween-20 (PBST), membranes were treated with secondary antibodies labeled with infrared fluorophores (LiCore Biosciences). After further washes in PBST, blots were imaged with the Odyssey infrared imaging system (LiCore Biosciences).

### Immunolabeling

Immunolabeling was performed as described previously [28]. Samples were de-paraffinized and antigens unmasked by heating in a pressure cooker to 95°C and 15 psi for 20 min in Trilogy alkaline solution with EDTA (Cell Marque), followed by 3 min in 90% formic acid. Samples were then blocked in PBS with 5% serum, species matched to secondary antibody. Tissue was incubated in rabbit polyclonal anti-GPX4 (1:800, AbFrontier) overnight at 4°C in 3% serum. After washes, sections were incubated in biotinylated secondary antibody followed by ABC™ reagent. HRP signals were developed with 3, 3-diaminobenzidine hydrochloride (DAB, Vector Labs), with or without the addition of nickel chloride to darken color as per manufacturer's instructions.

### Double Immunolabeling

Following first primary antibody, tissue was subsequently blocked in 5% normal horse serum, followed with separate blocking steps in streptavidin and biotin solutions (from ABC kit) five minutes each before second primary antibody reaction. Additional primary antibodies used were anti-α-synuclein (AS) (1:1000, Chemicon or 1:50, Abcam) and anti-tyrosine hydroxylase (TH, 1:8000, Sigma). Combinations of HRP-labeled secondary antibodies detected with DAB, DAB containing nickel chloride (DAB-Ni) or Nova Red reactions (Vector Laboratories), or alkaline phosphatase (AP) detected with BCIP reactions, were used to maximize contrast between the different antibodies.

### Fluorescent Immunolabeling

Depariffinization, antigen unmasking and primary antibody labeling were performed as described above. After washes, tissue was incubated in secondary antibodies conjugated to Alexa 488 and Alexa 546. Endogenous fluorescence was reduced by treating with an autofluorescence eliminator reagent (Chemicon).

### Spectral Imaging and Confocal Microscopy

Bright light and fluorescent images of midbrain tissue samples were imaged using an Olympus microscope equipped with the Nuance multispectral imaging system (Cambridge Research and Instrumentation, Inc). After obtaining spectral libraries for bright light images of unlabeled tissue and neuromelanin and fluorescent images of fluorophores and background autofluorescence, brightfield and fluorescent images were "unmixed" into individual signal components (i.e. neuromelanin or fluorescent probes) that were pseudocolored for comparison.

Confocal images were collected with a Zeiss LSM Pascal laser confocal microscope and analyzed with ImageJ software.

### Stereology

Volume-density of immunolabeling was determined with a Cavalieri probe using Stereologer software (Stereology Resource Center). First, the region (SN or putamen) was outlined using 5× magnification, and a computer-generated array of systematic-random loci were then visited and observed under 40×. A Cavalieri probe was placed over 50% of the image with an array of points (+), and the fraction of points contacting immunolabeled cells were used to estimate the area fraction of immunolabeling at that location. The total area fraction of immunolabeling was estimated as the average area fraction of all systematic-randomly chosen sites. According to the Delesse principle, area fraction on random sections is equivalent to the volume fraction [29].

## List of Abbreviations

AS: alpha synuclein; DA: dopamine; DAB: 3-diaminobenzidine hydrochloride; GPX: glutathione peroxidase; GPX1: glutathione peroxidase 1; GPX4: glutathione peroxidase 4 (phospholipid hydroperoxidase); HAAS: Honolulu-Asia Aging Study; PD: Parkinson's disease; PMI: post-mortem interval; Se: selenium; SN: substantia nigra; TH: tyrosine hydroxylase.

## Competing interests

The authors declare that they have no competing interests.

## Authors' contributions

FPB, GWR, LRW, and MJB designed the studies. ABM-B, AVR and TM aided with design detail and contributed essential interpretations of findings. MTB, AST and FPB performed the immunohistochemistry and FPB and ABM-B performed western blots. FPB, LAS and AVR performed microscopy and imaging. AST and LAS performed stereology. FPB wrote the paper with the assistance of all authors, who have read and approved the final manuscript.
